# Burden of disease in patients with Morquio A syndrome: results from an international patient-reported outcomes survey

**DOI:** 10.1186/1750-1172-9-32

**Published:** 2014-03-07

**Authors:** Christian J Hendriksz, Christine Lavery, Mahmut Coker, Sema Kalkan Ucar, Mohit Jain, Lisa Bell, Christina Lampe

**Affiliations:** 1 Manchester Academic Health Science Centre, The Mark Holland Metabolic Unit, Salford Royal Foundation NHS Trust, Salford, UK; 2 Society for Mucopolysaccharide Diseases, Buckinghamshire, UK; 3 Department of Pediatric Metabolism, Faculty of Medicine, Ege University, Izmir, Turkey; 4 BioMarin Europe Ltd, London, UK; 5 BioMarin Pharmaceutical Inc, Novato, CA, USA; 6 Department of Pediatric and Adolescent Medicine, Villa Metabolica, University Medical Center of the Johannes Gutenberg-University, Mainz, Germany

**Keywords:** Mucopolysaccharidosis IV, Morquio A syndrome, Patient-reported outcomes, Quality of life, Wheelchairs, Mobility, Pain measurement, Fatigue, EQ-5D-5L

## Abstract

**Background:**

Morquio A syndrome (or mucopolysaccharidosis IVa) is an ultra-rare multi-organ disease, resulting in significantly impaired functional capacity, mobility and quality of life (QoL).

**Methods:**

This patient-reported outcomes survey evaluated the global burden of Morquio A among adults (≥18 years, N = 27) and children (7-17 years, N = 36), including the impact on mobility, QoL, pain and fatigue. QoL was assessed using the general Health-Related Quality of Life (HRQoL) questionnaire (the EuroQol [EQ]-5D-5L). Pain and pain interference with daily activities were assessed using the Brief Pain Inventory Short Form (BPI-SF) in adults and the Adolescent Pediatric Pain Tool (APPT) in children. Fatigue was assessed by questioning the patients on the number of evenings in a week they felt extremely tired.

**Results:**

The clinical data showed a wide heterogeneity in clinical manifestations between patients, with the majority of patients showing differing levels of endurance, short stature, bone and joint abnormalities, abnormal gait and eye problems. Mobility was considerably impaired: 44.4% of children and 85.2% of adult patients were using a wheelchair. High wheelchair reliance significantly reduced QoL. This was mainly driven by reduced scores in the Mobility, Self-care, and Usual Activity domains. The HRQoL utility values were 0.846, 0.582 and 0.057 respectively in adults not using a wheelchair, using a wheelchair only when needed and always using a wheelchair; values were 0.534, 0.664 and –0.180 respectively in children. Employed adult patients had a better HRQoL than unemployed patients (HRQoL utility value 0.640 vs. 0.275, respectively).

64% of children and 74% of adult patients had joint pain; fatigue was reported by 69% of children and 63% of adults. Overall, increased mobility was associated with more severe and widespread pain and more fatigue.

**Conclusions:**

The HRQoL of Morquio A patients is mainly driven by the ability to remain independently mobile without becoming wheelchair dependent. Their QoL reduces dramatically if they always have to use their wheelchair. Even a slightly better mobility (wheelchair use only when needed) greatly improves QoL. Maintenance of functional capacity and mobility paired with better pain management are likely to improve QoL.

## Background

Morquio A syndrome, or mucopolysaccharidosis (MPS) IVa, is an ultra-rare, inherited lysosomal storage disorder caused by a deficiency in the enzyme *N*-acetylgalactosamine-6-sulfatase (GALNS). This leads to impaired degradation of the glycosaminoglycans (GAGs) chondroitin-6-sulfate (CS) and keratan sulfate (KS) [1,2] and disruption of the extracellular matrix function. Patients with Morquio A typically show widespread skeletal and joint abnormalities, including dwarfism with short trunk and neck, genu valgum, hip dysplasia, joint hypermobility, spinal abnormalities and pectus carinatum [3,4]. These skeletal abnormalities generally result in impaired endurance, walking ability and gait. Cardiopulmonary disease (including tracheal stenosis/malacia and dyspnoea) and spinal cord compression may further reduce endurance and/or mobility [5]. Many patients with Morquio A will sooner or later need walking aids or a wheelchair to assist with mobility [3,4]. The clinical manifestations of the disease and resulting impaired mobility can reduce the patient’s ability to perform activities of daily living (ADL) [4], such as attendance at school or work and social activities, and may have a considerable impact on the quality of life (QoL) of the patient, close relatives and caregivers. The patient’s QoL can be further compromised by frequent infections, impaired vision or hearing, frequent surgeries and (joint) pain and/or fatigue [3,4,6]. As Morquio A patients retain normal intelligence [6], adult patients are often employed and functioning in society, as long as physically capable. Therefore, reduced physical functioning/mobility may not only impact on the patient’s QoL but may also require a significant amount of care and increase the burden on society as a whole.

There is a tremendous variation between Morquio A patients, in terms of spectrum of clinical manifestations/organ systems involved, severity of manifestations and progression rate [6]. The life expectancy of patients with Morquio A also varies considerably, with some patients surviving until the second or third decade of life and others with near-normal life expectancy [4].

This patient-reported outcomes (PRO) survey was set-up to assess the global burden of disease among patients with Morquio A, including the impact on mobility/wheelchair use, HRQoL, pain and fatigue and the interaction between these factors.

## Methods

### Study design and patient selection

The study was an international, voluntary, single-assessment, cross-sectional paper-based survey administered in person or by mail via local staff members of MPS patient advocacy/support groups and/or physicians and clinics. The study took place from June 2012 to April 2013.

The study population consisted of Morquio A patients and their caregivers identified by patient advocacy/support groups and/or physicians/clinics from Brazil, Colombia, Germany, Spain, Turkey and the UK. These countries have strong patient advocacy/support groups and a fair number of patients. Eligible patients had to be ≥ 7 years of age (some exceptions were made due to the limited number of patients) and treatment-naïve, i.e. not on enzyme replacement therapy. Caregivers (≥18 years old) who served as the primary caregiver of at least one patient with Morquio A were recruited after enrolment of a family member with Morquio A in the survey. Eligible patients and caregivers had to be able to speak, write and understand both the verbal and written language of their country. All were informed on all pertinent aspects of the study and were willing to participate and complete the surveys. All patients and caregivers who met the study entry criteria and completed the survey were included in the analysis. The low number of UK patients in this cohort can be explained by the fact that many of the patients were part of the different enzyme replacement therapy clinical trials and the fact that the phase 1/2 enzyme replacement therapy study was performed in the UK only.

### Assessments

Two survey versions for patients, one for adult patients (≥ 18 years) and one for "children" (≤ 17 years old), and one caregiver survey were used. If a patient was unable to physically complete the surveys, the survey administrators recorded verbatim responses from the patient. Child surveys were completed by the patient with assistance from a parent or carer, if necessary. Ethics approval was obtained in Germany and all patients (or their caregivers) signed an informed consent/ascent form. No ethics approval was required in the other countries, due to the survey nature of the study.

The patient surveys were developed through review of the existing literature to understand the clinical and humanistic impacts of Morquio A and to identify the most relevant generic, disease-specific and symptom-specific PROs. They consisted of stand-alone and validated HRQoL questionnaires. Stand-alone questions were developed jointly by BioMarin Europe Ltd, the UK MPS Society and some clinical experts treating Morquio A patients. Pilot questionnaires were assessed among adult patients in the UK through two focus groups who judged their clarity, relevance and value. Additionally, the groups identified important questions that were not included in the questionnaire and questions that could be deleted to reduce respondent burden. The questionnaires were revised and finalised based on the feedback obtained. The questionnaires were developed in English and translated and culturally adapted to each targeted country. These translated and culturally-adapted versions of validated questionnaires for target patients were obtained from instrument developers, when possible.

The final patient questionnaires began with stand-alone questions concerning demographics. Generic HRQoL and pain were assessed using validated PRO measures. Generic HRQoL was assessed using the General Health-Related Quality of Life EuroQoL(EQ)-5D-5L questionnaire [7]. This is a generic standardised measure of health status developed by the EuroQoL group and applicable to a wide range of health conditions and therapies. It comprises five dimensions (5D): Mobility, Self-care, Usual activities, Pain/Discomfort and Anxiety/Depression. EQ-5D health states can be converted into a single summary index value (utility) by applying a formula that essentially attaches weights to each of the levels in each dimension. This formula is based on the valuation of EQ-5D health states from general population samples [7]. A HRQoL utility value of "1" represents perfect health; a value of "0" represents death. Subsequent normalisation to a healthy population can provide negative values indicating that the patient is feeling worse than death. Pain and pain interference in daily life were assessed using the Brief Pain Inventory Short Form (BPI-SF) in adult patients [8] and the Adolescent Pediatric Pain Tool (APPT) [9] in children. Fatigue was assessed by questioning the patients on the number of evenings in a week they were feeling extremely tired. More detailed information regarding these validated questionnaires is available as Additional file 1.

The caregiver questionnaire contained questions concerning demographics, family relationships and social characteristics, and self-reported time spent on caregiving. The present paper will only shortly discuss results regarding self-reported time spent on caregiving.

### Statistical analysis

The present paper presents the outcomes of the demographic and clinical characteristics of the patients and the outcomes of the validated PRO measures and caregiver time.

Frequency distributions (number and percentage of patients in each category) were used for categorical variables and descriptive statistics (mean, median, standard deviation/error [SD/SE], minimum and maximum scores) for continuous and count variables. Responses to open-ended questions were listed. For validated instruments, scores were calculated according to the instrument’s scoring guidelines. For more information, see Additional file 1.

Unpaired (type 3) t-tests were used to calculate *P*-values for differences in outcomes between the following mobility groups in adults and children: patients never using a wheelchair vs. patients using a wheelchair only when needed vs. patients always using a wheelchair.

## Results

### Demographics and clinical characteristics

Completed questionnaires were received from 27 adults and 36 children. The largest number of respondents (38%) came from Germany (Additional file 2). Of the adult patients, about half (52%) were between ages 18 and 24 years. The vast majority of adult patients (85%) lived with their parents, while 7.4% lived alone. Practically all patients (92-96%) showed short stature (Additional file 3) with a mean height in adult patients of 115.1 cm (Additional file 2). Bone deformity was present in approximately three in four patients, while abnormal gait was more prevalent in adult patients (96%) than in children (75%). Other common clinical manifestations, occurring in ≥40% of adults or children, were joint pain, difficulty in joint movement/joint stiffness or joint laxity/hypermobility, eye problems, fatigue/low stamina, difficulty breathing, cervical spine instability, hearing loss and dental problems. In both adults and children, joint laxity was most common at the wrists, fingers, ankles, knees and elbows; joint stiffness was particularly common in adults, mostly in the shoulders and back or cervical spine.

Additional file 2 gives information on the mobility of the patients. Thirty % of adults and 25% of children used a walking aid, most commonly crutches or a walker in adults and a walker, braces, ankle foot orthoses or splints in children. Wheelchair use increased with age, with most adult patients (85%) using a wheelchair. In adults, 39% of wheelchair users used their wheelchair always and 61% used it only when needed; in children, 13% of wheelchair users used it always and 88% used it only when needed.

### HRQoL vs. mobility/wheelchair use and employment status

**Figure 1:**
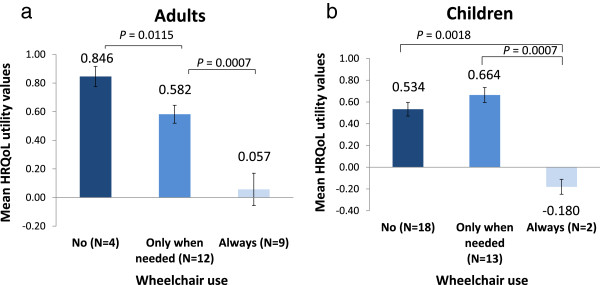
**Health-related quality of life (HRQoL) measured using the EQ5D-5L questionnaire in adults (a) and children (b) with Morquio A according to wheelchair use/mobility level.** A score of 1 indicates "perfect health", a score of 0 indicates "death". Negative values indicate "feeling worse than death". Presented as mean value and standard error of the mean.

Analysis of the EQ-5D-5L questionnaire showed that in both adult patients and children the HRQoL was most negatively affected in the domains Mobility, Self-care and Usual activities (Additional file 4). Adults who did not use a wheelchair had a statistically significantly better HRQoL than those who used a wheelchair only when needed, 0.846 vs. 0.582 (*P* = 0.0115; Figure 1a), respectively. This was not observed in children (Figure 1b). Patients who always used their wheelchair, both adults and children, reported statistically significantly lower HRQoL than those who use their wheelchair only when needed (0.057 vs. 0.582 for adults and-0.180 vs. 0.664 for children, both *P* = 0.0007; Figure 1), respectively. The finding that Self-care is heavily affected in adult patients using a wheelchair always was also confirmed by caregivers, showing that average caregiving time for these patients is much higher (13.8 hours during weekdays) than for those not using a wheelchair (1.3 hours) or using a wheelchair only when needed (3.9 hours; Additional file 5).

**Figure 2:**
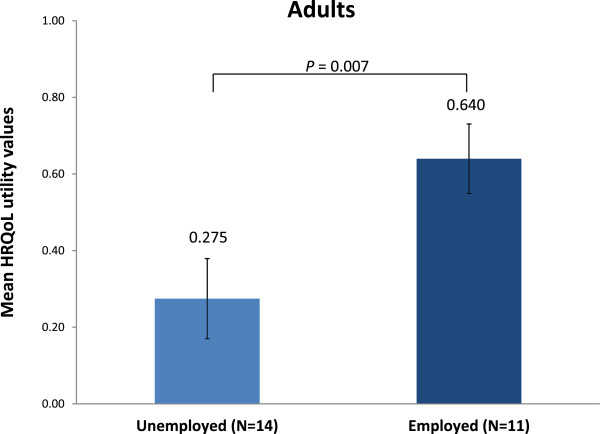
**Health-related quality of life (HRQoL) measured using the EQ5D-5L questionnaire in adult patients with Morquio A according to employment status.** A score of 1 indicates "perfect health", a score of 0 indicates "death". Negative values indicate "feeling worse than death". Presented as mean value and standard error of the mean.

Of the 27 adult patients, 14 were unemployed and 12 were employed; employment status was missing for 1 patient. Unemployed adult patients had a statistically significantly worse HRQoL than employed patients (Figure 2). Employed patients were more likely to be active and mobile; 67% of employed patients used a wheelchair vs. 100% of unemployed patients; 2 of the 9 patients (22%) who always used a wheelchair were employed vs. 6 of the 13 patients (50%) using their wheelchair only when needed.

### Pain, pain interference and fatigue vs. mobility/wheelchair use

**Figure 3:**
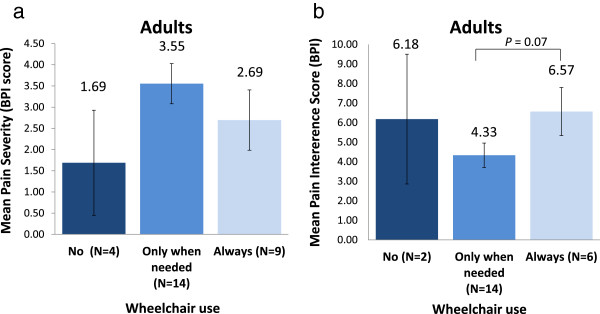
**Pain severity (a) and pain interference with daily activities (b) in adults with Morquio A according to wheelchair use/mobility level.** Pain severity and pain interference were evaluated using the Brief Pain Inventory (BPI); 1 = no pain/no pain interference, 10 = worst pain ever/complete pain interference. Pain interference scores were unavailable for 2 of the 4 patients in the "no wheelchair" and for 3 of the 9 patients in the "always wheelchair" groups. Presented as mean score and standard error of the mean.

Joint pain was experienced by 74% of adult patients and 64% of children (Additional file 3). This was related to wheelchair use and intensity of wheelchair use, with slightly different results between adults and children. In adults, non-wheelchair users had milder pain, took less pain medication and/or had less body parts affected by pain than patients who used a wheelchair (Figure 3a and Additional file 6). Within the group of wheelchair users, those who used their wheelchair always reported pain less frequently, had milder pain and used less pain medication than those who use their wheelchair only when needed (Figure 3a and Additional file 6). More mobile wheelchair users (using a wheelchair only when needed) also reported more widespread pain (i.e. in more body parts) than patients using their wheelchair always (of whom most patients reported pain around the lower extremities) (Additional file 6). However, in adult wheelchair users, pain interference scores reduced with increased mobility, with patients always using a wheelchair having the greatest pain interference with daily activities (Figure 3b). In children, those who used their wheelchair always had less severe pain (mean APPT pain severity score of 1.00?±?SE 1.00) than those who did not use a wheelchair (4.38?±?0.72, *P* = 0.049) or used their wheelchair only when needed (4.00?±?0.75, *P* = 0.054). The child non-wheelchair users did not only have numerically the highest pain severity, but also (slightly) more frequently reported pain across different parts of the body than wheelchair users, including the spinal area (63% vs. 57% of patients), lower extremities (100% vs. 93% of patients), upper extremities (69% vs. 43% of patients) and head and neck area (56% vs. 43% of patients).

Fatigue/low stamina was reported frequently by both adults (63%) and children (69%; Additional file 3). The impact of wheelchair use on fatigue and/or energy level followed the same pattern as for pain: better mobility was associated with more fatigue. Adults who always used their wheelchair had higher energy levels (i.e. lower proportion of patients feeling extremely tired on 1 or more evenings per week) than those who used their wheelchair only when needed (Additional file 7). The energy level in the adults that did not use a wheelchair was in between that of those using a wheelchair always and those using it only when needed. The results in children were comparable. None of the children always using a wheelchair felt extremely tired on 1or more evenings per week as compared to the majority of children who were more mobile (Additional file 7).

## Discussion

The clinical features reported in this international PRO survey support the findings in natural history studies that Morquio A is a progressive, multi-organ/systemic, heterogeneous disease manifesting not only in the musculoskeletal system (as bone and joint abnormalities) but also affecting the eyes, ears, teeth and (cardio)respiratory system [3,4]. The most frequently reported clinical manifestations, occurring in the majority of patients, were short stature, bone deformity, joint abnormality (both laxity and stiffness), eye problems, cervical spine instability and respiratory problems. The clinical characteristics of the children included in the present survey are in line (Additional file 8) with those from 2 large multi-national natural history studies, i.e. the International Morquio A Registry [3] and the MorCAP study [4]. The International Morquio A Registry included 326 patients with a median age of 11 years for males and 16 years for females [3]. The MorCAP study included 325 patients with a median age of 11.6 years [4]. The mean height of the children in this survey was 109.9 cm (range 84 to 162.5 cm) compared to 104.2 cm (range 77.8 to 180.2 cm) in the MorCAP study [4]. The mean height of the adults in this survey was 115.1 cm (± standard deviation [SD] of 25.8) compared to 122.5 (± SD 22.5) cm in men and 116.5 (± SD 20.5) cm in women over 18 years old in the International Registry and compared to 176.2 (± SD 6.1) cm and 163.1 (± SD 5.4) cm in healthy controls at 18 years [3].

The survey confirmed that these multi-systemic clinical manifestations generally lead to abnormal gait (in 75-96% of patients) and increasing disability, with patients becoming increasingly dependent on walking aids and, in particular, a wheelchair when they get older. Forty-four % of children used a wheelchair, which increased to 85% in adults. In the natural history studies, respectively 31% [3] and 45% [4] used a wheelchair. The increasing disability and reliance on a wheelchair has a major impact on the patients’ independence, illustrated by the fact that the vast majority (85%) of the adult patients in this survey still lived with their parents.

The multi-systemic clinical manifestations of the patients included in the survey were not only associated with increasing disability and reduced mobility, they also caused pain across a range of body parts and fatigue in most patients.

Taking into account the debilitating nature of Morquio A, it is not surprising that the patients included in the survey had a reduced HRQoL as measured by the validated EQ-5D-5L questionnaire versus the general population. HRQoL was affected considerably more in some patients than in others, reflecting the variability in their ability to remain independently mobile, i.e. to move without using a wheelchair. Whereas the HRQoL utility value in adult non-wheelchair users was still relatively good (0.846), it was statistically significantly reduced if patients had to use a wheelchair and approached a feeling of death (0.057) when the wheelchair was used always. This negative impact of wheelchair use on HRQoL was mostly driven by worse scores for the domains Mobility, Self-care and Usual activities. The great impact of wheelchair use on self-care was confirmed by the finding that the dependence of patients on caregivers increased with increasing wheelchair use. The average number of caregiving hours for adult patients increased from 1.3 hours on a weekday in non-wheelchair users to 3.9 hours if the wheelchair was used only when needed and up to 13.8 hours (practically all waking hours of the day) when the wheelchair was used always. Another factor that may have influenced the very low HRQoL in the patients who had to use their wheelchair always is employment status. The survey showed that adult patients who had to use their wheelchair always had a very high chance of being unemployed, which was associated with a lower HRQoL than being employed. The impact of wheelchair use on QoL was also shown in a study of 210 adults with Pompe disease which used the Short-Form (SF) 36 to measure HRQoL [10]. In this study, wheelchair users had significantly lower physical and social functioning scores than non-wheelchair users.

The validated EQ-5D-5L has been used in many other (chronic) disease areas to evaluate HRQoL. The mean HRQoL utility value of the adult patients in this survey who used their wheelchair only when needed (0.582) was worse than that reported for patients with chronic ischaemic heart disease (0.640) or non-insulin dependent diabetes mellitus (0.670) [11] and only slightly better than that of patients with moderate to severe rheumatoid arthritis (RA; 0.489) [12] or with multiple sclerosis (MS) requiring a walking aid (0.460) [13]. The HRQoL of the adult Morquio A patients who always needed a wheelchair in this survey (0.057) was only slightly better than that of MS patients who are bed ridden or completely immobile (-0.049) [13]. Arne et al. who evaluated HRQoL in patients with chronic diseases such as chronic obstructive pulmonary disease (COPD), RA and diabetes mellitus also concluded that physical inactivity increases the burden of disease [14].

The most mobile adult patients (i.e. the non-wheelchair users) demonstrated better HRQoL versus patients who used a wheelchair. This was accompanied by lower pain severity scores on the BPI-SF. The adult patients using a wheelchair only when needed had more severe and more widespread pain than the patients always using a wheelchair but their pain interference score was lower. This suggests that adult Morquio A patients try to tolerate pain as long as independence (from their wheelchair and caregivers) and mobility are retained. However, once pain affects independence, there is a steep decrease in QoL. As the pain interference analysis only included 2 adult patients not using a wheelchair, no conclusions can currently be made regarding pain interference in this group. In the adult patient group, the EQ-5D Pain/Discomfort domain scores did not follow the pain severity scores obtained by the BPI-SF for the different mobility categories. Most likely, this is due to the fact that the Pain/Discomfort score of the EQ-5D-5L is based on a single question whereas the BPI-SF comprises different questions to assess pain severity and will be able to capture more subtle differences between patients.

The results for children using a wheelchair were in line with those in adults using a wheelchair. Children who used their wheelchair always experienced less severe and less widespread pain and were less frequently feeling extremely tired at the end of the day than those who used their wheelchair only when needed. However, unlike what was seen in adults, HRQoL in children using a wheelchair only when needed was better than in children not using a wheelchair. This is probably due to the fact that children not using a wheelchair had more severe pain (reflected in both the APPT pain severity score and the EQ-5D-5L Pain/Discomfort domain score) and pain across more body parts than those using a wheelchair only when needed. The fact that adults who always used a wheelchair had more severe pain than children and were more often feeling extremely tired at the end of the day may reflect the progression of the disease over time/with age.

This PRO survey is the first study in patients with Morquio A to show that although using a wheelchair all of the time reduces pain severity and distribution and tiredness at the end of the day due to limited functional activity, it also considerably reduces HRQoL. Therefore, efforts should be made to maintain as much as possible the ability to be independently mobile (e.g. by pain management and appropriate use of walking aids) in these patients while managing their energy level. Studies in patients with MS showed that greater walking and mobility problems were associated with higher unemployment rates, increased requirement for caregiver support and higher healthcare resource utilisation and that interventions improving mobility could have a significant impact on patients, caregivers and society as a whole [15]. Findings from these studies on MS are directly applicable to that of the Morquio A patient population studied here. It should be noted that recruiting through patients advocacy/support groups and physicians may have resulted in recruitment bias in the present study. Also, the results of the present survey should be interpreted in light of the relatively low number of patients included (even for this ultra-rare disease) and the facts that for some assessments information was missing for some patients and the questionnaires were not validated specifically for Morquio A. Nevertheless, even with this low patient number, the difference in QoL between patients with different levels of mobility was statistically significant, suggesting a very strong association between wheelchair use and QoL. The next critical evaluation will be to try and equate this information with currently used clinical trial outcome measures like the 6-minute walk test/distance (6MWT/6MWD). This has been done for Duchenne muscular dystrophy and has shown that when looking at heterogeneous disorders the improvements in 6MWT will be very variable [16]. This study concluded that "At lower levels of function, smaller increases in 6MWD result in a meaningful change in quality of life (QoL) instrument scores. At higher levels of function, larger increases may be necessary to achieve the same QoL change score".

## Conclusions

The HRQoL of Morquio A patients is mainly driven by the ability to remain independently mobile without using a wheelchair. QoL is dramatically reduced in patients who use their wheelchair always. Even slightly better mobility (i.e. using a wheelchair only when needed) improves QoL dramatically. Although wheelchair use can considerably reduce pain, clinicians should be aware of the detrimental impact on the patient’s QoL and should not encourage patients to go down this route too readily. Maintenance of functional capacity and mobility paired with better pain management are likely to improve QoL.

## Competing interests

Chris Hendriksz: Financial support has been received in person or by the institution from BioMarin in the following capacities: honoraria for lectures, chairman of advisory boards, consultant on projects, research trials and travel grants. Christine Lavery: Financial support has been received in person or by the Society for Mucopolysaccharide Diseases from BioMarin in the following capacities: participant on advisory board, patient access programme for clinical trials, travel grants and unrestricted educational grants. Mahmut Coker: The author declares that he has no competing interests. Sema Kalkan Ucar: The author declares that she has no competing interests. Mohit Jain: Receiving financial support/salary as employee of BioMarin. Lisa Bell: Receiving financial support/salary as employee of BioMarin. Christina Lampe: Received speaker’s and consultant honoraria, travel support and unrestricted grants from Shire, BioMarin and Genzyme.

## Authors’ contributions

CH: Contributed to questionnaire design and gave advice on results interpretation and analysis. CL: Contributed to project, questionnaire and focus group design, reached out to UK patients and gave advice on interpretation and analysis. MC & SKU: Contributed by gaining feedback from Turkish patients. MJ: Contributed as project lead across the whole project. LB: Contributed as project sponsor to project and questionnaire design. CL: Contributed to questionnaire design, led the ethics approval review, reached out to German patients and gave advice on interpretation and analysis. All authors reviewed the manuscript for important intellectual content and approved the final manuscript.

## Supplementary Material

Additional file 1 **Validated patient reported outcomes measures.** Document including detailed information regarding the validated questionnaires used in the study. Click here for file

Additional file 2 **Patient demographics and mobility.** Data show number (%) of patients. Percentages may not add up to 100% due to rounding. Table showing information on age, height, gender, country, other family members with Morquio A, use of walking aids and wheelchair for children and adult patients included in the study. Click here for file

Additional file 3 **Clinical characteristics occurring in at least 40% of children or adults.** Table showing clinical characteristics occurring in at least 40% of children or adults included in the study. Click here for file

Additional file 4 **Mean score for the five EQ-5D-5L domains according to mobility/wheelchair use.** Data for adults and children with Morquio A. Table showing mean score for the five EQ-5D-5L domains (Mobility, Self-care, Usual activities, Pain/Discomfort, Anxiety/Depression) in adults and children with Morquio A not using a wheelchair, patients only using a wheelchair when needed, and patients always using a wheelchair. Click here for file

Additional file 5 **Mean number of caregiving hours/day on weekdays and weekends for adults and children with Morquio A, according to wheelchair use/mobility level.** Table showing the mean number of caregiving hours/day on weekdays and weekends in adults and children with Morquio A according to wheelchair use/mobility level. Mobility levels compared in adults: no wheelchair, wheelchair only when needed, wheelchair always; mobility levels compared in children: no wheelchair use, wheelchair use. Click here for file

Additional file 6 **Proportion of adult patients experiencing pain, using pain medication and number and location of body parts affected by pain according to mobility/wheelchair use.** Table showing the proportion of adult patients experiencing pain, using pain medication and number and location of body parts affected by pain according to mobility/wheelchair use. Click here for file

Additional file 7 **Proportion of adults (a) and children (b) with Morquio A feeling extremely tired 0, 1-2 or ≥ 3 evenings per week according to mobility/wheelchair use.** Graph showing the proporation of adults and children with Morquio A feeling extremely tired 0, 1-2 or ≥ 3 evenings per week according to mobility/wheelchair use. Click here for file

Additional file 8 **Comparison of clinical manifestations and need for walking aids or wheelchair use in children enrolled in this survey vs. those enrolled in natural history studies **[3,4]**.** Graph showing clinical manifestations and need for walking aids or wheelchair use in children enrolled in this survey vs. those enrolled in natural history studies. Click here for file
