# Supplementary material for: Burden of disease in patients with Morquio A syndrome: results from an international patient-reported outcomes survey
Source: Orphanet J Rare Dis. 2014 Mar 7;9:32. doi: 10.1186/1750-1172-9-32 (PMC4016149; doi:10.1186/1750-1172-9-32)
Supplement: Additional file 1 — Validated patient reported outcomes measures. Document including detailed information regarding the validated questionnaires used in the study. [file 1750-1172-9-32-S1.docx]

**Supplementary material 1: Validated patient reported outcomes measures**

The questionnaires were designed to analyze a host of outcomes measures from study subjects. No validated, disease-specific HRQoL measures exist for Morquio A. Therefore, the measures described below, which have been validated across a wide number of diseases, were used to assess general HRQoL and impact of pain among patients.

**General Health-Related Quality of Life: EQ-5D-5L**

Subjects’ overall HRQoL was measured by using the EQ-5D-5L, which is a generic standardized measure of health status developed by the EuroQoL group and applicable to a wide range of health conditions and therapies. It provides a simple descriptive profile and a single index value for health status. The instrument is designed for self-completion by respondents and takes approximately 3-5 minutes to complete.

The EQ-5D-5L consists of two components - the EQ-5D descriptive system and the EQ visual analogue scale (EQ VAS). The descriptive system comprises five dimensions: mobility, self-care, usual activities, pain/discomfort and anxiety/depression. Each dimension has five levels coded from 1 to 5: no problems (1), slight problems (2), moderate problems (3), severe problems and unable to function (mobility, self-care and usual activities; 4) or extreme problems (pain/discomfort and anxiety/depression; 5). Subject response to this component results in a 1-digit number expressing the level selected for each dimension. The digits for five dimensions can be combined into a 5-digit number describing the respondent’s health state.

Respondents indicate their self-rated health on the EQ VAS, a 20 cm vertical, visual analogue scale numbered from 0 to 100 where the endpoints are labelled ‘Best health you can imagine’ (100) and ‘Worst health you can imagine’ (0). This information can be used as a quantitative measure of health outcome as judged by the individual respondents.

Finally, EQ-5D health states, defined by the descriptive system, may be converted into a single summary index value (utility) by applying a formula that essentially attaches weights to each of the levels in each dimension. This formula is based on the valuation of EQ-5D health states from general population samples. A HRQoL utility value of “1” represents perfect health; a value of “0” represents death. Subsequent normalisation to a healthy population can provide negative values indicating that the patient is feeling worse than death.

**Pain: Brief Pain Inventory and Adolescent Pediatric Pain Tool**

The Brief Pain Inventory Short Form (BPI-SF), one of the most widely used measurement tools to assess clinical pain, was employed with adult patients. Patients rated the severity and location of their pain and the impact on daily functioning. Use of pain medications and amount of pain relief is also documented. The BPI-SF takes approximately five minutes to complete.

The Adolescent Pediatric Pain Tool (APPT), a validated tool to evaluate pain in children and adolescents, was used to assess pain among children. Respondents indicated the location(s) of their pain and the severity of their pain and selected words to describe their pain.
