# Supplementary material for: Burden of disease in patients with Morquio A syndrome: results from an international patient-reported outcomes survey
Source: Orphanet J Rare Dis. 2014 Mar 7;9:32. doi: 10.1186/1750-1172-9-32 (PMC4016149; doi:10.1186/1750-1172-9-32)
Supplement: Additional file 2 — Patient demographics and mobility. Data show number (%) of patients. Percentages may not add up to 100% due to rounding. Table showing information on age, height, gender, country, other family members with Morquio A, use of walking aids and wheelchair for children and adult patients included in the study. [file 1750-1172-9-32-S2.docx]

**Supplementary material 2: Patient demographics and mobility.**Data show number (%) of patients. Percentages may not add up to 100% due to rounding

|  | **Children** | **Adults** |
| --- | --- | --- |
| **N** | 36 | 27 |
| **Age in years: N (%)**  **5-9**  **10-14**  **15-17**  **18-24**  **25-29**  **30-34**  **35-39**  **≥40** | 11 (30.6)  17 (47.2)  8 (22.2)  0  0  0  0  0 | 0  0  0  14 (51.9)  5 (18.5)  2 (7.4)  3 (11.1)  3 (11.1) |
| **Mean height: cm** | 109.9 | 115.1 |
| **Gender: N (%)**  **Male**  **Female** | 20 (55.6)  16 (44.4) | 15 (55.6)  12 (44.4) |
| **Country: N (%)**  **Brazil**  **Colombia**  **Germany**  **Spain**  **Turkey**  **United Kingdom** | 7 (19.4)  10 (27.8)  10 (27.8)  6 (16.7)  2 (5.6)  1 (2.8) | 4 (14.8)  4 (14.8)  14 (51.9)  3 (11.1)  2 (7.4)  0 |
| **Other family members with Morquio A: N (%)**  **Yes**  **No**  **Missing** | 15 (41.7)  20 (55.6)  1 (2.8) | 14 (51.9)  13 (48.1)  0 |
| **Using walking aids: N (%)**  **Using a wheelchair: No; N (%) Using a wheelchair: Yes; N (%)**  **Only when needed**  **Always** | 9 (25.0)  20 (55.6)  16 (44.4)  14 (38.9)  2 (5.6) | 8 (29.6)  4 (14.8)  23 (85.2)  14 (51.9)  9 (33.3) |
