# Supplementary material for: Burden of disease in patients with Morquio A syndrome: results from an international patient-reported outcomes survey
Source: Orphanet J Rare Dis. 2014 Mar 7;9:32. doi: 10.1186/1750-1172-9-32 (PMC4016149; doi:10.1186/1750-1172-9-32)
Supplement: Additional file 3 — Clinical characteristics occurring in at least 40% of children or adults. Table showing clinical characteristics occurring in at least 40% of children or adults included in the study. [file 1750-1172-9-32-S3.docx]

**Supplementary material 3**: **Clinical characteristics occurring in at least 40% of children or adults**

| **% of patients with:** | **Children  (N = 36)** | **Adults  (N = 27)** |
| --- | --- | --- |
| **Short stature** | 91.7 | 96.3 |
| **Bone deformity** | 72.2 | 77.8 |
| **Abnormal gait** | 75.0 | 96.3 |
| **Joint pain** | 63.9 | 74.1 |
| **Joint laxity/hypermobility**  **Wrists**  **Ankles**  **Fingers**  **Elbows**  **Knees** | 88.9  72.2  69.4  66.7  38.9  33.3 | 66.7  66.7  48.1  55.6  40.7  40.7 |
| **Joint stiffness**  **Shoulders**  **Spine/cervical spine** | 52.8  22.2  22.2 | 77.8  55.6  44.4 |
| **Cervical spine instability** | 52.8 | 48.1 |
| **Eye problems** | 63.9 | 74.1 |
| **Fatigue/low stamina** | 69.4 | 63.0 |
| **Respiratory Problems** | 44.4 | 51.9 |
| **Hearing loss** | 36.1 | 48.1 |
| **Dental problems** | 72.2 | 37.0 |
