# Supplementary material for: Burden of disease in patients with Morquio A syndrome: results from an international patient-reported outcomes survey
Source: Orphanet J Rare Dis. 2014 Mar 7;9:32. doi: 10.1186/1750-1172-9-32 (PMC4016149; doi:10.1186/1750-1172-9-32)
Supplement: Additional file 4 — Mean score for the five EQ-5D-5L domains according to mobility/wheelchair use. Data for adults and children with Morquio A. Table showing mean score for the five EQ-5D-5L domains (Mobility, Self-care, Usual activities, Pain/Discomfort, Anxiety/Depression) in adults and children with Morquio A not using a wheelchair, patients only using a wheelchair when needed, and patients always using a wheelchair. [file 1750-1172-9-32-S4.docx]

**Supplementary material 4:** **Mean score for the five EQ-5D-5L domains according to mobility/wheelchair use.**Data for adults and children with Morquio A

| **Mean EQ-5D-5L score for domain** | **Wheelchair use:** | | | All patients |
| --- | --- | --- | --- | --- |
|  | **No** | **Only when needed** | **Always** |  |
| **Adults: N** | 4 | 13 | 9 | 26 |
| - Mobility | 1.75 | 3.29 | 4.89 | 3.59 |
| - Self-care | 1.25 | 2.50 | 4.22 | 2.89 |
| - Usual activities | 1.25 | 2.93 | 4.11 | 3.07 |
| - Pain/Discomfort | 2.00 | 2.62 | 2.67 | 2.54 |
| - Anxiety/Depression | 1.75 | 2.33 | 1.78 | 2.04 |
| **Children: N** | 20 | 13 | 2 | 35 |
| - Mobility | 2.47 | 2.77 | 5.00 | 2.74 |
| - Self-care | 2.50 | 2.62 | 5.00 | 2.69 |
| - Usual activities | 2.35 | 2.33 | 3.50 | 2.41 |
| - Pain/Discomfort | 2.50 | 1.92 | 1.00 | 2.20 |
| - Anxiety/Depression | 1.70 | 1.85 | 1.00 | 1.71 |

Each dimension was coded from 1 to 5: no problems, slight problems, moderate problems, severe problems, and unable to function (mobility, self-care and usual activities) or extreme problems (pain/discomfort and anxiety/depression) (see also Supplementary material 1)
