# Supplementary material for: Burden of disease in patients with Morquio A syndrome: results from an international patient-reported outcomes survey
Source: Orphanet J Rare Dis. 2014 Mar 7;9:32. doi: 10.1186/1750-1172-9-32 (PMC4016149; doi:10.1186/1750-1172-9-32)
Supplement: Additional file 5 — Mean number of caregiving hours/day on weekdays and weekends for adults and children with Morquio A, according to wheelchair use/mobility level. Table showing the mean number of caregiving hours/day on weekdays and weekends in adults and children with Morquio A according to wheelchair use/mobility level. Mobility levels compared in adults: no wheelchair, wheelchair only when needed, wheelchair always; mobility levels compared in children: no wheelchair use, wheelchair use. [file 1750-1172-9-32-S5.docx]

**Supplementary material 5:** **Mean number of caregiving hours/day on weekdays and weekends for adults and children with Morquio A, according to wheelchair use/mobility level**

| **Average caregiving time (hours/day)** | **Wheelchair use:** | | |
| --- | --- | --- | --- |
|  | **No** | **Only when needed** | **Always** |
| **Caregivers of adults with Morquio A: N** | 2 | 5 | 9 |
| - Weekdays | 1.3 | 3.9 | 13.8 |
| - Weekends | 1.8 | 4.1 | 14.3 |
|  | **No** | **Yes** | |
| **Caregivers of children with Morquio A: N** | 19* | 15** | |
| - Weekdays | 12.0 | 12.1 | |
| - Weekends | 15.1 | 14.0 | |

*N = 18 for weekends; **N = 14 for weekdays
