# Supplementary material for: Burden of disease in patients with Morquio A syndrome: results from an international patient-reported outcomes survey
Source: Orphanet J Rare Dis. 2014 Mar 7;9:32. doi: 10.1186/1750-1172-9-32 (PMC4016149; doi:10.1186/1750-1172-9-32)
Supplement: Additional file 6 — Proportion of adult patients experiencing pain, using pain medication and number and location of body parts affected by pain according to mobility/wheelchair use. Table showing the proportion of adult patients experiencing pain, using pain medication and number and location of body parts affected by pain according to mobility/wheelchair use. [file 1750-1172-9-32-S6.docx]

**Supplementary material 6: Proportion of adult patients experiencing pain, using pain medication and number and location of body parts affected by pain according to mobility/wheelchair use**

| **Wheelchair use:** | **No (N = 4)** | **Only when needed  (N = 14)** | **Always  (N = 9)** |
| --- | --- | --- | --- |
| **Patients experiencing pain, %** | 50 | 86 | 44 |
| **Patients experiencing moderate to severe pain^1^, %** | 25 | 43 | 33 |
| **Patients experiencing moderate to severe  pain interference^2^, %** | 50 | 50 | 67 |
| **Patients on pain medication, %** | 50 | 64 | 44 |
| **Mean number of body parts affected by pain^3^** | 0.3 | 4.2 | 2.0* |
| **Patients experiencing pain in different body parts, %^3^** |  |  |  |
| - **Spinal area** |  | 64% | 29% |
| - **Lower extremities** |  | 93% | 86% |
| - **Upper extremities** |  | 57% | 29% |
| - **Head and neck area** |  | 57% | 14% |

Pain severity and pain interference were evaluated using the Brief Pain Inventory (BPI);
1 = no pain / no pain interference, 10 = worst pain ever / complete pain interference.
^1^Moderate to severe pain defined as Pain Severity Score > 4

^2^Moderate to severe pain interference defined as Pain Interference Score > 5
^3^ N=7 for “always wheelchair” group; location of pain for the different body parts was available for only 1 patient in the “no wheelchair” group and therefore data are not shown

**P* < 0.001 vs. only when needed
