# Supplementary material for: Burden of disease in patients with Morquio A syndrome: results from an international patient-reported outcomes survey
Source: Orphanet J Rare Dis. 2014 Mar 7;9:32. doi: 10.1186/1750-1172-9-32 (PMC4016149; doi:10.1186/1750-1172-9-32)

Supplementary material 7

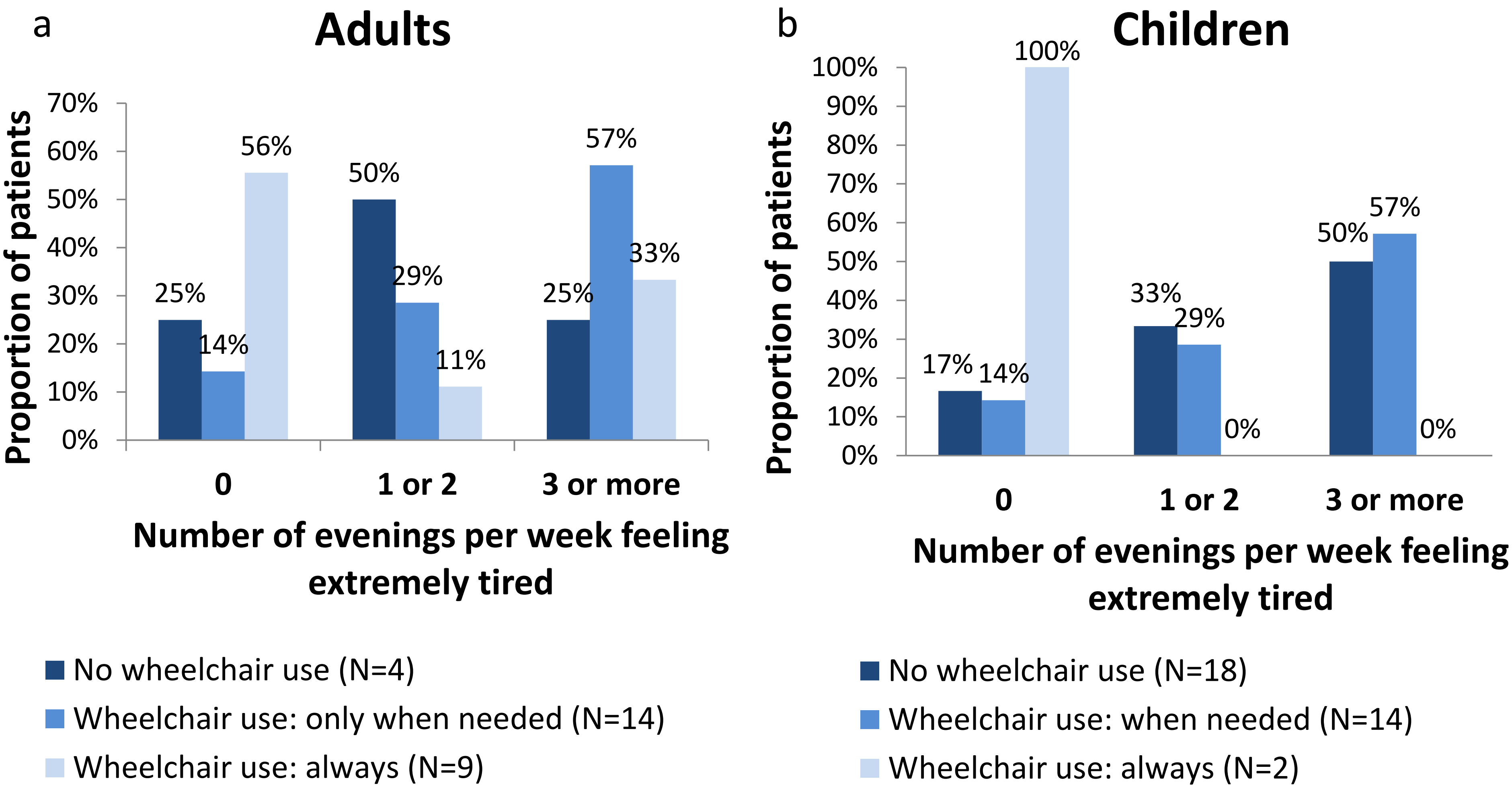

Supplement: Additional file 7 — Proportion of adults (a) and children (b) with Morquio A feeling extremely tired 0, 1-2 or ≥ 3 evenings per week according to mobility/wheelchair use. Graph showing the proporation of adults and children with Morquio A feeling extremely tired 0, 1-2 or ≥ 3 evenings per week according to mobility/wheelchair use. [file 1750-1172-9-32-S7.pdf]
