# Supplementary figures and images for: Burden of disease in patients with Morquio A syndrome: results from an international patient-reported outcomes survey
Source: Orphanet J Rare Dis. 2014 Mar 7;9:32. doi: 10.1186/1750-1172-9-32 (PMC4016149; doi:10.1186/1750-1172-9-32)

Supplementary material 8

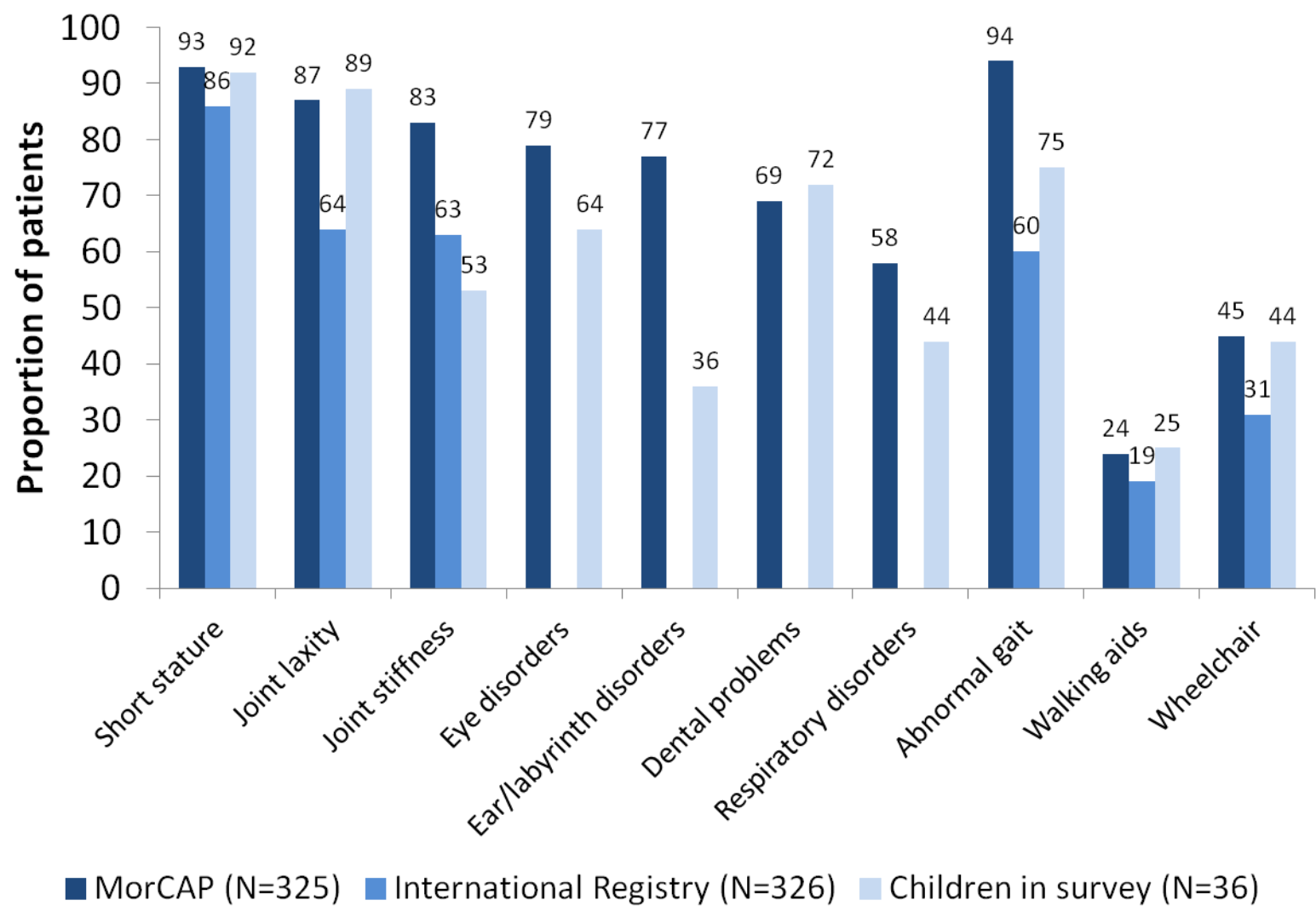

Supplement: Additional file 8 — Comparison of clinical manifestations and need for walking aids or wheelchair use in children enrolled in this survey vs. those enrolled in natural history studies [3,4]. Graph showing clinical manifestations and need for walking aids or wheelchair use in children enrolled in this survey vs. those enrolled in natural history studies. [file 1750-1172-9-32-S8.pdf]
